# DeepPPAPredMut: deep ensemble method for predicting the binding affinity change in protein–protein complexes upon mutation

**DOI:** 10.1093/bioinformatics/btae309

**Published:** 2024-05-08

**Authors:** Rahul Nikam, Sherlyn Jemimah, M Michael Gromiha

**Affiliations:** Department of Biotechnology, Bhupat and Jyoti Mehta School of Biosciences, Indian Institute of Technology Madras, Chennai 600036, India; Department of Biotechnology, Bhupat and Jyoti Mehta School of Biosciences, Indian Institute of Technology Madras, Chennai 600036, India; Department of Biomedical Engineering, Khalifa University, P.O. Box: 127788 , Abu Dhabi, United Arab Emirates; Department of Biotechnology, Bhupat and Jyoti Mehta School of Biosciences, Indian Institute of Technology Madras, Chennai 600036, India; Department of Computer Science, Tokyo Tech World Research Hub Initiative (WRHI), Institute of Innovative Research, Tokyo Institute of Technology, 4259 Nagatsutacho, Midori-ku, Yokohama, Kanagawa 226-8501, Japan

## Abstract

**Motivation:**

Protein–protein interactions underpin many cellular processes and their disruption due to mutations can lead to diseases. With the evolution of protein structure prediction methods like AlphaFold2 and the availability of extensive experimental affinity data, there is a pressing need for updated computational tools that can efficiently predict changes in binding affinity caused by mutations in protein–protein complexes.

**Results:**

We developed a deep ensemble model that leverages protein sequences, predicted structure-based features, and protein functional classes to accurately predict the change in binding affinity due to mutations. The model achieved a correlation of 0.97 and a mean absolute error (MAE) of 0.35 kcal/mol on the training dataset, and maintained robust performance on the test set with a correlation of 0.72 and a MAE of 0.83 kcal/mol. Further validation using Leave-One-Out Complex (LOOC) cross-validation exhibited a correlation of 0.83 and a MAE of 0.51 kcal/mol, indicating consistent performance.

**Availability and implementation:**

https://web.iitm.ac.in/bioinfo2/DeepPPAPredMut/index.html.

## 1 Introduction

Protein–protein interactions (PPIs) are essential for many biological processes, such as cell signaling, metabolism, and gene expression (Gromiha 2020b). The binding affinity of protein–protein complexes are dictated by various factors such as favorable noncovalent interactions (Gromiha 2020a), composition of amino acids at the interface for thermal adaptation (Ma *et al.* 2010) as well as conformational properties, ⍺-helical and β-strand tendencies, bulkiness, and the number of aromatic and charged residues (Yugandhar and Gromiha 2014a,b). Mutations in protein–protein complexes may disturb the functionality and affect the stability of the complex, leading to diseases (Nishi *et al.* 2013, Petukh *et al.* 2015, Pandey and Gromiha 2023). Although several experimental techniques are available to study the effect of mutation on binding affinity (change in binding free energy upon mutation, ΔΔG), these methods are labor-intensive, time-consuming, and expensive. Hence, several computational methods have been developed to predict the binding free energy changes upon mutation in protein–protein complexes (Gromiha *et al.* 2017). The available methods are broadly categorized into two classes based on input parameters, i.e. structure-based and sequence-based methods.

Structure-based methods such as FoldX (Guerois *et al.* 2002, Schymkowitz *et al.* 2005), MutaBind (Li *et al.* 2016), BindProfX (Xiong *et al.* 2017), mCSM-PPI2 (Rodrigues *et al.* 2019), iSee (Geng *et al.* 2019), MutaBind2 (Zhang *et al.* 2020), SAAMBE-3D (Pahari *et al.* 2020), and DLA-Mutation (Behbahani *et al.* 2023) use the structural properties from protein complexes to predict the change in binding affinity upon mutation. The utility of the structure-based methods is limited by the availability of relatively fewer experimentally known structurers of protein–protein complexes compared to sequence information.

Further, sequence-based methods have been developed for predicting changes in binding affinity upon mutation. ProAffiMuSeq is the first sequence-based prediction method, focusing on mutations situated at the interface (Jemimah *et al.* 2020). Subsequently, SAAMBE-SEQ introduced a gradient-boosting decision tree model incorporating changes in physicochemical properties to predict alterations in binding affinity (Li *et al.* 2021). Despite these advancements, leveraging the information on comprehensive experimental datasets, sophisticated computational models of protein structures such as AlphaFold2 (Jumper *et al.* 2021) and RoseTTAFold (Baek *et al.* 2021), and deep learning methods enhance the efficiency and performance of predicting the changes in binding affinity caused by mutations.

In this work, we have addressed all these limitations and developed a deep ensemble model, DeepPPAPredMut, which takes a protein sequence and mutation as input from the user and predicts the change in the binding affinity upon mutation in the protein complex. Our method uses physicochemical properties, PSSM, and amino acid properties. In addition, we have used graph-based properties for mutation sites, such as the degree of the residue and hydrogen bond donor/acceptor of the residue. Our model showed a correlation and MAE of 0.83 and 0.51 kcal/mol, respectively in a set of 2591 mutants.

## 2 Materials and methods

### 2.1 Dataset

To develop the prediction model, we utilized experimentally known binding affinity data collected from PROXiMATE (Jemimah *et al.* 2017) and SKEMPIv2 (Jankauskaite *et al.* 2019) and the literature. The SKEMPIv2 and PROXiMATE databases contain 7085 and 6293 single mutations from 348 and 173 protein–protein complexes, respectively and 3345 mutations in 131 complexes are common between them. Further, we have collected 473 mutations from 65 proteins from the literature. Initially, we consolidated data from these resources and excluded proteins with >25% sequence identity. Next, we refined the dataset by eliminating redundant mutations. Further, all the mutations, which are present in the validation dataset (see below) are removed from this dataset. The final dataset is comprising of 2591 mutations from 236 proteins. For data with binding affinity on dissociation constant (*K*_d_), we calculated the binding free energy (Δ*G*) using the following formula:

(1) $$\Delta G=-RT\ln\left(1/K_{d}\right)$$

*R*: gas constant; *T*: temperature in Kelvin; *K*_d_: binding affinity of the complex. Further, to calculate the change in binding free energy upon mutation (ΔΔ*G*), we used the following equation:

(2) $$\Delta \Delta G={\Delta G}_{\mathrm{mut}}-{\Delta G}_{\mathrm{wild}}$$

ΔG_mut_ and ΔG_wild_ are binding free energies of mutant and wild-type protein–protein complex, respectively.

For validation purposes and to compare the model performance with existing state-of-the-art methods, we utilized the benchmark (validation) dataset created by Geng *et al.* (2019), which is used in other studies (Jemimah *et al.* 2020, Li *et al.* 2021, Behbahani *et al.* 2023). It includes 19 mutations from NM dataset for interleukin4-receptor complex (Benedix *et al.* 2009), S487 dataset with 487 mutations in 56 protein complexes (Jankauskaite *et al.* 2019) and 33 mutations in MDM2-p53 complex (Geng *et al.* 2019). The combined dataset contains a total of 473 mutations in 65 complex structures, which are not used for training and cross-validation. Supplementary Fig. S1 represents the distribution of ΔΔ*G* in our dataset and benchmark dataset.

**Figure 1.**
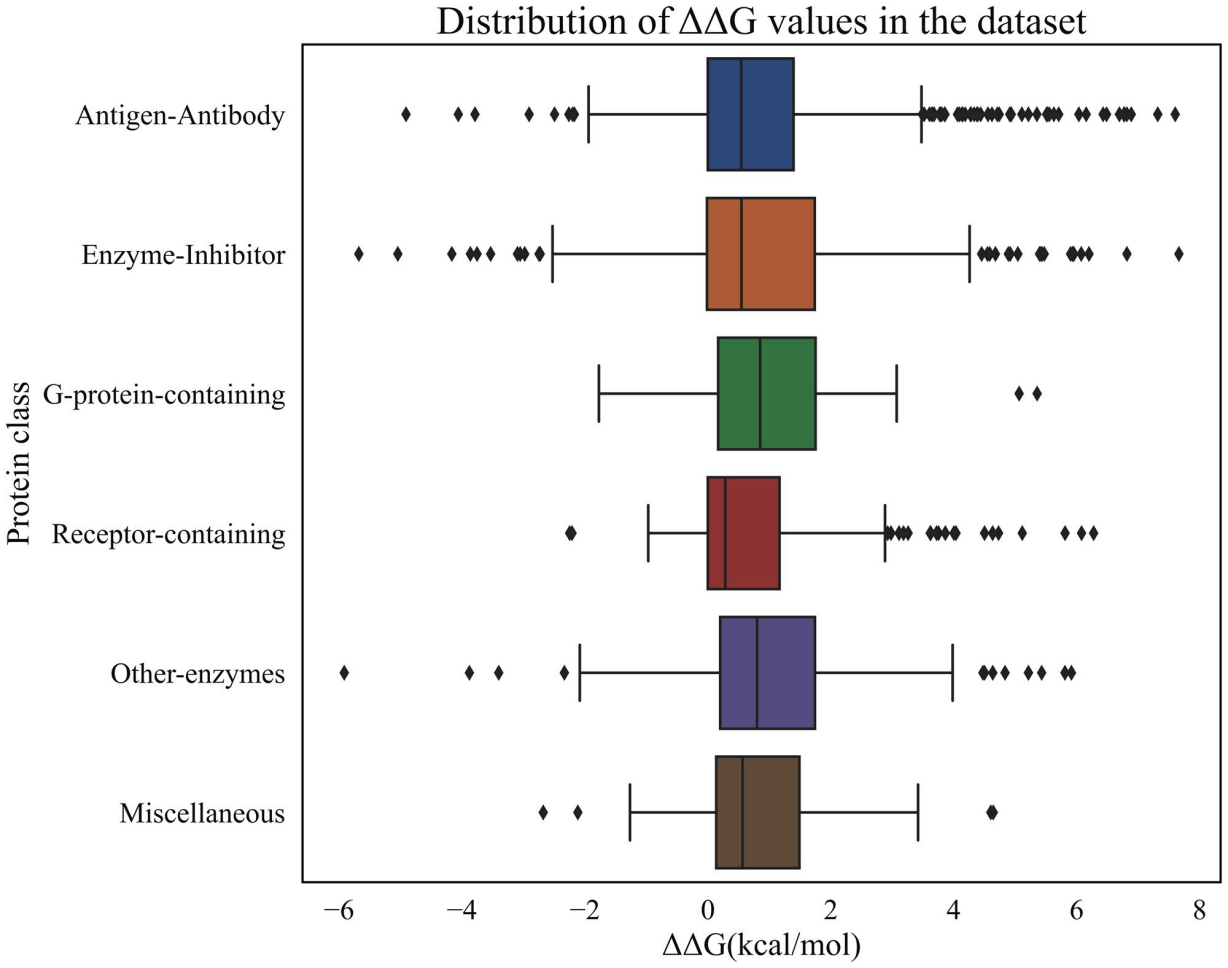
Distribution of ΔΔG values across different protein functional classes.

In our earlier studies, we observed that the binding affinity of protein–protein complexes depends on functional classes of proteins, which have different ranges of binding free energies (Yugandhar and Gromiha 2014a,b, Jemimah and Gromiha 2018, Nikam *et al.* 2022). Further, the classification of mutants based on functional classes of proteins enhanced the prediction performance (Jemimah *et al.* 2020). Hence, we divided the dataset into six subsets based on the functional class of each protein–protein complex: (i) antigen-antibody, (ii) enzyme-inhibitor, (iii) G-protein-containing, (iv) receptor-containing, (v) other-enzyme containing, and (vi) miscellaneous. The distribution of ΔΔ*G* values for individual protein functional classes is summarized in Fig. 1. We have noted a wide range of ΔΔ*G* values across different classes. Specifically, receptor-containing complexes has the lowest ΔΔ*G* value of −2.25 kcal/mol, whereas the class “other-enzyme” has the lowest ΔΔ*G* of −3.75 kcal/mol. Enzyme-inhibitor complexes have ΔΔ*G* values, ranging from −5.91 to 7.66 kcal/mol, with an average value of 0.95 kcal/mol. On the other hand, G-protein complexes have the range of −1.78 to 5.33 kcal/mol with a similar average ΔΔ*G*.

### 2.2 Feature calculation

To develop the model, we calculated various sequence and structure-based features. We considered the predicted structures from the AlphaFold database (Varadi *et al.* 2022) to calculate structure-based features.

#### 2.2.1 Sequence-based features

We used a set of 103 amino acid properties (Supplementary Table S1), which are shown to be relevant for protein folding studies (Chaudhary *et al.* 2015). In addition, we calculated protein interface-based features for each amino acid from PIFACE (Cukuroglu *et al.* 2014), which are reported to be important to understand mutational effects on protein–protein complexes (Jemimah *et al*. 2020), PSSM matrix and conservation score using AACon (Manning *et al.* 2008, Valdar 2002). Further, we included mutation-based features such as net volume, flexibility of the residue, change in hydrophobicity, and fluctuation of amino acid upon mutation. These features have been used in the literature for predicting the effect of mutation on stability, folding rate and binding affinity (Pahari *et al.* 2020, Li *et al.* 2021, Gromiha 2024). The change in property value (Δ*P*) is computed as the difference between each property value of mutant (*P*_mut_) and wild-type (*P*_wild_) residues.

#### 2.2.2 Structure-based features

To compute structure-based features, we utilized the predicted structures reported in the AlphaFold database (Varadi *et al.* 2022). With these predicted structures, we computed various amino acid features, such as solvent-accessible area, using Naccess (Hubbard and Thornton 1993), number of hydrogen bond acceptors and donors using HBPLUS (McDonald and Thornton 1994) and residue depth, using a python script adapted from the Biopython module. Furthermore, we calculated graph-based features for the mutation site, such as degree centrality, betweenness centrality, eigenvalue centrality, and closeness centrality. To compute graph-based features, we designed a graph in which each node corresponds to an amino acid. Edges are established between pairs of amino acids when their spatial distance is <8 Å. These features provide information regarding the connectivity of the mutated site, which could be helpful in predicting the change in binding affinity. In addition, we considered mutation position (core, interior, support, rim, and surface) as defined by Levy (2010).

#### 2.2.3 Feature selection

Initially, we considered a set of 295 sequence and structure-based properties, which are relevant to protein–protein interactions. Further, we removed the highly correlated properties using a correlation coefficient cut-off > 0.85, which resulted in 90 properties. In addition, we utilized the “recursive feature elimination” from scikit-learn (Pedregosa *et al.* 2011) to select the topmost 50 important features. These selected features are used as input to the individual model development for each functional class.

#### 2.2.4 Deep learning model

In this study, we developed a deep ensemble model to predict the effect of mutations on binding affinity. Deep learning is a division of machine learning that works by connecting a number of neurons in multiple layers to capture the nonlinear relationship between input features and the change in binding free energy (ΔΔ*G*). In our previous work, we demonstrated the potential of deep learning to predict binding site residues accurately and to predict binding affinity ([Bibr btae309-B25][Bibr btae309-B24]).

**Figure 2.**
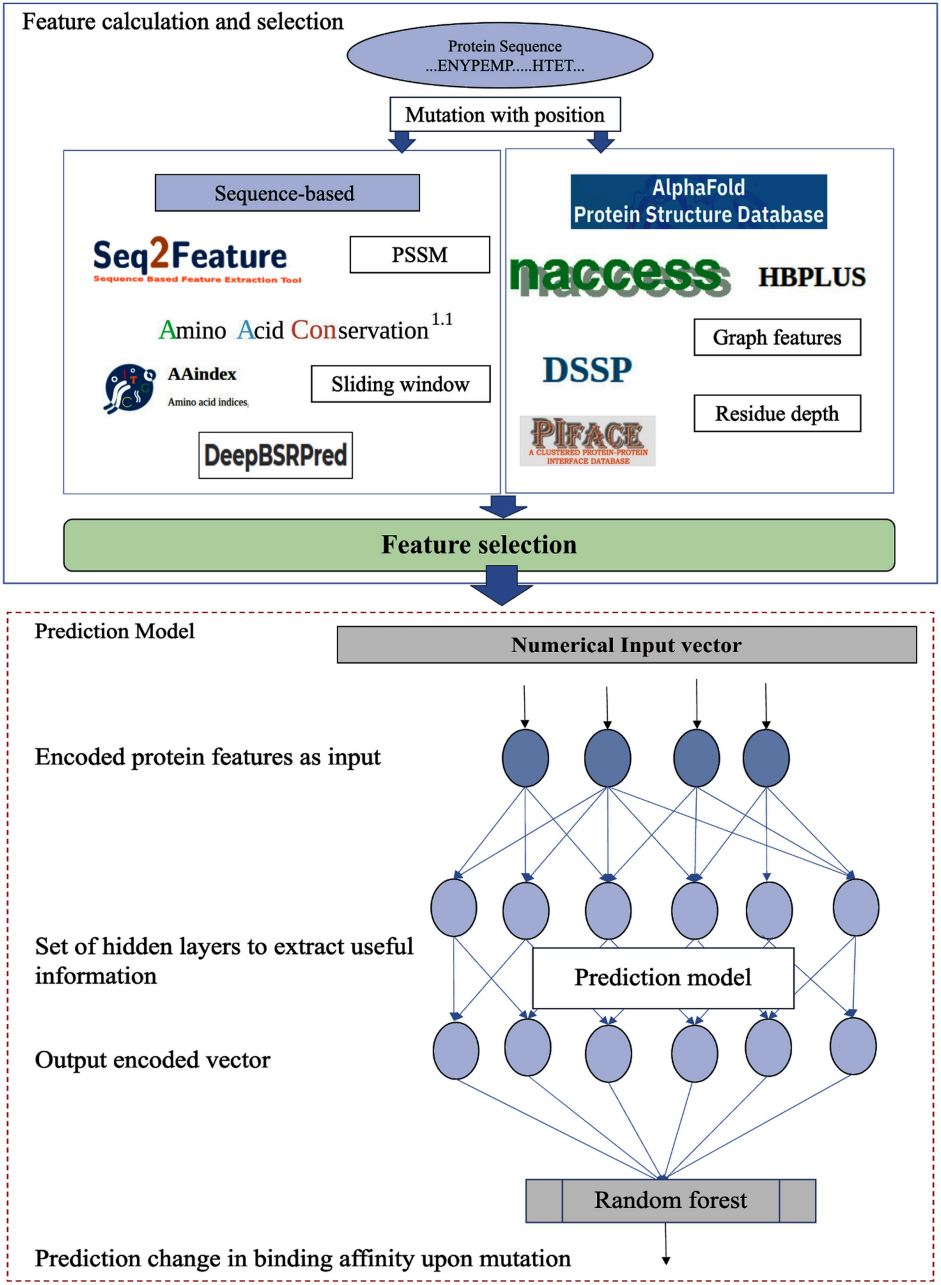
Overall workflow of the model.

Initially, we provided various curated features obtained after feature selection step to a Keras sequential model (https://keras.io/), which consists of three layers (Input, hidden, and output layers). The input layer takes the calculated features and passes them to the hidden layer which has Relu as activation function, which, in turn, passes them to the output layer. We have adapted the hyperparameter optimization through grid search and the dimensionality of the output layer is optimized to 12. This 12D encoded vector serves as the input for the random forest model. We chose the random forest model as our final predictor due to its superior performance compared to other machine learning methods. A detailed comparison between the random forest and other approaches is provided in Supplementary Table S2. The overall structure of the prediction model is depicted in Fig. 2.

#### 2.2.5 Performance validation

We utilized the 10-fold cross-validation technique to validate the performance of the method. The dataset was divided into 10 subsets, and during each iteration, 9 subsets were used for training, while one subset was used for testing the performance. This process was repeated 10 times to ensure reliable evaluation.

In addition, we used leave-out-one-complex cross-validation. In this approach, we removed mutations associated with one complex from the dataset and trained the model on the remaining data. Then, we tested the performance on the removed complex. We repeated this process for all available proteins in the dataset. This method allowed us to observe how well the model generalizes to unknown mutations in a new complex. Furthermore, the performance of the method has been assessed using the following measures:

(3) $$\mathrm{PCC}=n\Sigma xy-\left(\Sigma x\Sigma y\right)/\;\sqrt{n\Sigma x^{2}-\Sigma {\left(x\right)}^{2}}\sqrt{n\Sigma y^{2}-\Sigma {\left(y\right)}^{2}}$$

(4) $$\mathrm{MAE}=\Sigma \left|{\Delta \Delta G}_{\exp}-{\Delta \Delta G}_{\mathrm{pred}}\right|/n$$

*PCC* or Pearson's correlation coefficient is denoted by *R*. *MAE* stands for mean absolute error, and ΔΔ*G*_exp_ (*x*) and ΔΔ*G*_pred_ (*y*) represent experimental and predicted ΔΔ*G* values, respectively; *n* is the number of data.

## 3 Results and discussion

### 3.1 Predicting the change in binding affinity upon mutation

We utilized the deep ensemble model for predicting the change in binding affinity upon mutation using various sequence and structure-based features as input (see Section 2). We classified the overall dataset into subsets based on their functions, which is shown to be important for improving the prediction performance (Yugandhar and Gromiha 2014a,b, Jemimah *et al.* 2020, Nikam *et al.* 2022).

**Figure 3.**
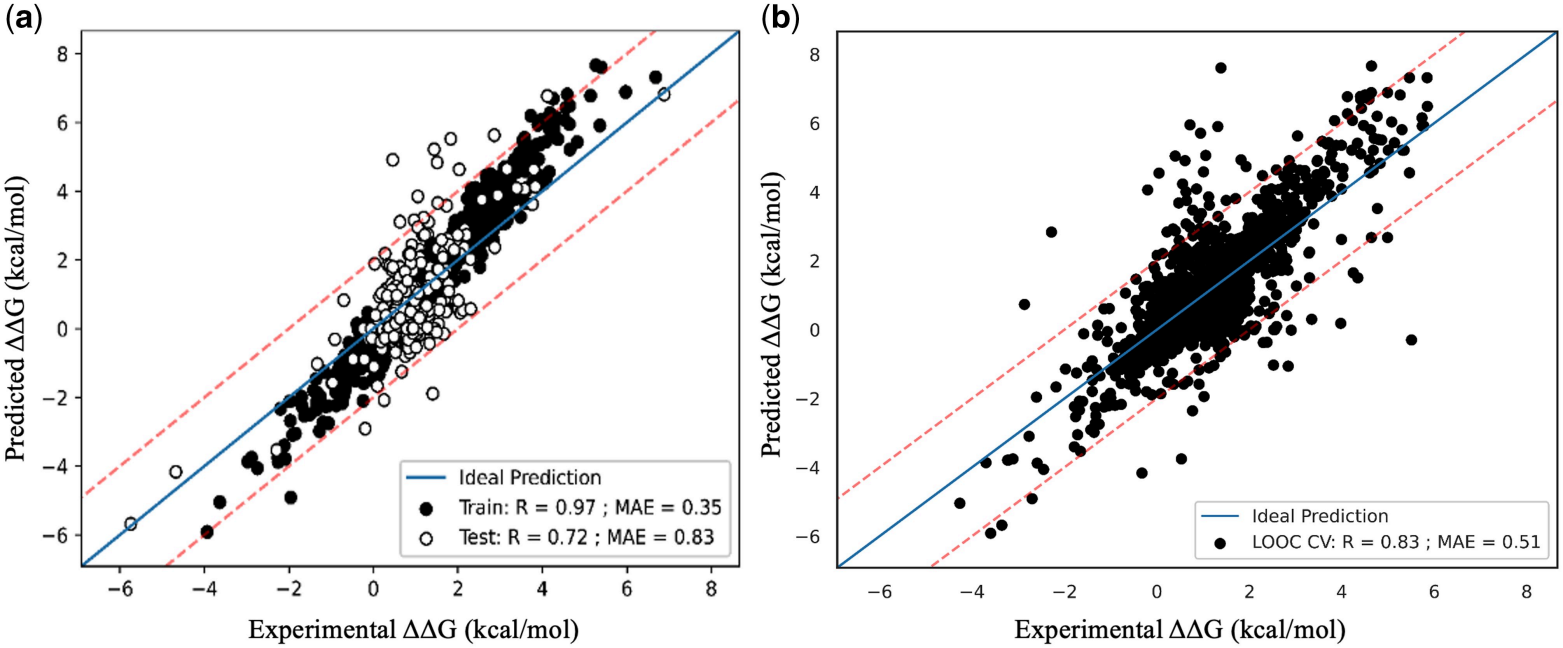
Performance of the present method on (a) training and test, and (b) LOOC cross validation.

The overall prediction performance of the method for training/test and LOOC cross validation is plotted in Fig. 3a and b, respectively. Our model achieved a correlation of 0.97 with a MAE of 0.35 on training set and a correlation of 0.72 with a MAE of 0.83 on test set. Further, our model showed consistent overall performance on LOOC cross validation with a correlation of 0.83 and a MAE of 0.51.

### 3.2 Prediction based on the protein functional class

**Figure 4.**
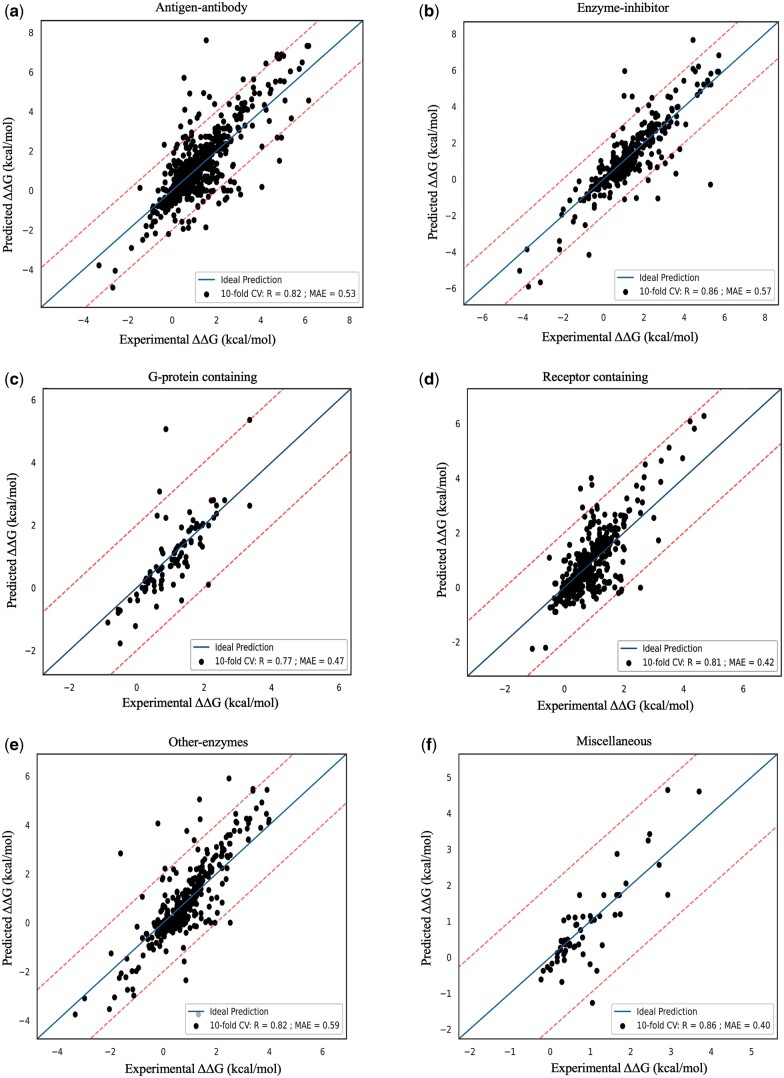
Prediction performance on individual functional class on 10-fold cross validation: (a) Antigen-antibody, (**b**) Enzyme-inhibitor, (**c**) G-protein containing, (**d**) Receptor containing, (**e**) Other-enzymes and (**f**) Miscellaneous.

We evaluated the prediction performance for each protein functional class, and the results obtained with 10-fold cross validation along with overall performance (mean of all protein functional classes) is summarized in Table 1 along with number of features used in each class. Figure 4 shows the relationship between experimental and predicted ΔΔG values.

**Table 1.** Performance of the model on individual functional class using 10-fold cross validation.

Functional class (no. of features)	Correlation	MAE (RMSE) kcal/mol
Antigen-antibody (20)	0.82	0.53 (0.67)
Enzyme-inhibitor (20)	0.86	0.57 (0.88)
G-protein-containing (19)	0.77	0.47 (0.52)
Receptor-containing (20)	0.81	0.42 (0.51)
Other-enzyme containing (20)	0.82	0.59 (0.62)
Miscellaneous (14)	0.86	0.40 (0.46)
Overall	0.83	0.51 (0.61)

#### 3.2.1 Antigen–antibody

The antigen-antibody dataset comprises 1032 mutations, and the experimental ΔΔ*G* values span from −4.91 to 7.6 kcal/mol. Our model has demonstrated an impressive performance, achieving a correlation coefficient of 0.82 with a MAE of 0.53 kcal/mol during 10-fold cross-validation (Fig. 4a). In addition, in the LOOC cross-validation scenario, our model achieved a correlation coefficient of 0.83 with a MAE of 0.51 kcal/mol (Supplementary Fig. S2a).

#### 3.2.2 Enzyme–inhibitor

Within this subset, there are 484 mutations, and the experimental ΔΔ*G* values vary from −5.91 to 7.66 kcal/mol. Our model displayed robust and reliable performance in both 10-fold and LOOC cross-validation, achieving a correlation coefficient of 0.86 with a MAE of 0.57 kcal/mol (Fig. 4b, Supplementary Fig. S2b).

#### 3.2.3 G-protein-containing

The G-protein dataset encompasses 94 mutations for which experimental ΔΔG data are available and it ranges from −1.78 to 5.33 kcal/mol. Remarkably, our model demonstrated a consistent performance in both 10-fold and LOOC cross-validation, yielding a correlation coefficient of 0.77 with a MAE of 0.47 kcal/mol (Fig. 4c, Supplementary Fig. S2c).

#### 3.2.4 Receptor-containing

The receptor-containing complexes involve interactions between receptors and various ligands, including growth hormone, insulin, interleukin, viral proteins, plasminogen activator, and lysozyme. Within this category, the experimental ΔΔG values span from −2.25 to 6.27 kcal/mol. Our model has showed consistent results, achieving a correlation coefficient of 0.81 and a MAE of 0.42 kcal/mol during 10-fold cross-validation, as depicted in Fig. 4d. Similarly, in LOOC, our model maintains a correlation of 0.81 with a MAE of 0.41 kcal/mol, as illustrated in Supplementary Fig. S2d.

#### 3.2.5 Other-enzyme containing

Within this specific category, enzymes form complexes with noninhibitor proteins. The experimental ΔΔG values for this set of interactions span from −3.75 to 5.90 kcal/mol. Our model demonstrated a correlation coefficient of 0.82 with a MAE of 0.59 kcal/mol during 10-fold cross-validation (Fig. 4e). In the LOOC scenario, the model maintained a strong correlation of 0.78 with a slightly increased MAE of 0.62 kcal/mol (Supplementary Fig. S2e).

#### 3.2.6 Miscellaneous

The miscellaneous class includes protein complexes that do not fall into any of the previously mentioned categories. Within our dataset, there are 100 mutations, and the experimental ΔΔ*G* values range from −1.26 to 4.64 kcal/mol. Our model showed a strong performance, with a correlation coefficient of 0.86 between the experimental and predicted ΔΔ*G* values and a MAE of 0.40 kcal/mol on10-fold cross-validation as depicted in Fig. 4f. Similarly, in LOOC, the model maintains a correlation of 0.82 with a MAE of 0.44 kcal/mol, as shown in Supplementary Fig. S2f.

Further, we examined the performance of the method using two additional evaluation procedures such as (i) cross validations are performed randomly at the level of variations, allowing two different substitutions occurring at the same site to be placed in the training and testing set, respectively and (ii) splits are performed such that mutations occurring at the same site are all confined either in training or testing sets. The results obtained with these two scenarios are presented in Supplementary Table S3. We observed that overall correlation is 0.86 and 0.82, respectively, along with a MAE of 0.49 and 0.50 kcal/mol.

### 3.3 Importance of selected features

We utilized SHapley Additive exPlanations (SHAP) plots as a tool to gain insights into the significance of individual features selected by our method across all functional classes (Lundberg *et al.* 2018). In Supplementary Fig. S3a–f, we have visualized and presented the contributions of these important features for each specific functional class.

We observed that certain features play a prominent role in influencing the predictions. Notably, graph-based features like closeness centrality, degree centrality, and eigenvector centrality consistently exhibit substantial contributions across various functional classes. These features reflect the structural and connectivity aspects of the protein network and appear to be crucial in explaining the variations in ΔΔ*G* values due to mutations.

In addition, we have found that other factors, such as the residue position within the protein, the hydrophobicity of the mutated residue at the mutation site, and the change in volume due to mutation, also make significant contributions. These features provide valuable information about the local environment and the physical changes induced by mutations, contributing to our understanding of the underlying mechanisms that affect binding affinity.

In summary, our SHAP plot analysis reveals that a combination of structural and biochemical features, including graph-based properties and residue-specific characteristics, plays a pivotal role in predicting the impact of mutations on binding affinity across different functional classes. These insights enhance our comprehension of the factors influencing protein interactions and mutation effects.

### 3.4 Performance comparison on blind datasets

**Figure 5.**
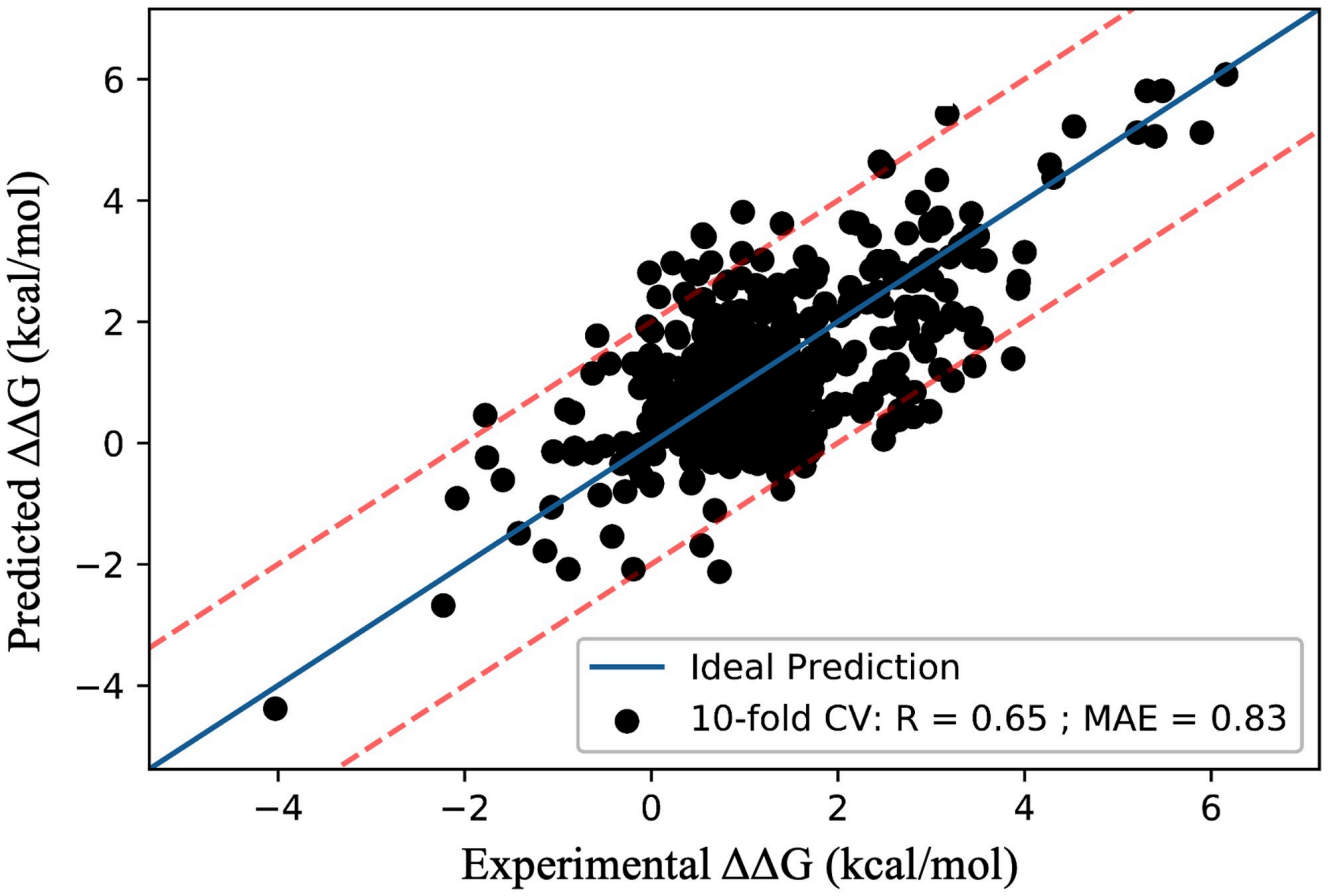
Prediction performance on benchmark dataset.

To construct the validation dataset, we amalgamated mutations and predictions from three sources: the NM dataset, the S487 dataset, and the MDM_p53 dataset. This unified dataset comprises a total of 473 mutations extracted from 65 complex structures, distinct from those employed in the training and test datasets. It encompasses a wide range of ΔΔ*G* values, spanning from −4.38 to 6.08 kcal/mol, and comprehensively represents all functional classes. Our model achieved a correlation of 0.65 with a MAE of 0.83 kcal/mol on this benchmark dataset and the performance is consistent in all the three datasets. The overall performance of the model on the benchmark dataset is represented in Fig. 5. In addition, a comparison with existing methods is presented in Table 2. From Table 2, it is evident that our method (DeepPPAPredMut) outperforms other methods in the literature.

**Table 2.** Comparison of DeepPPAPredMut with other existing methods.^a^

Method	Reference	R	RMSE	MAE kcal/mol
iSEE	Geng *et al.* (2019)	0.26	1.30	0.99
FoldX	Guerois *et al.* (2002)	0.35	1.50	1.07
mCSM	Rodrigues *et al.* (2019)	0.25	1.34	0.96
BindProfX	Xiong *et al.* (2017)	0.39	1.21	0.91
SAAMBE-3D	Pahari *et al.* (2020)	0.39	1.20	0.92
ProAffiMuSeq	Jemimah *et al.* (2020)	0.27	1.41	1.06
SAAMBE-SEQ	Li *et al.* (2021)	0.35	1.31	1.01
DLA-Mutation	Behbahani *et al.* (2023)	0.48	1.14	0.88
DeepPPAPredMut	Present work	0.65	1.11	0.83

a The PCC and MSE for iSee, FoldX, mCSM, BindProfX, ProAffiMuSeq, are taken from Jemimah *et al.* (2020) and SAAMBE-3D, SAAMBE-SEQ is taken from Li *et al.* (2021).

### 3.5 Webserver

We have developed a user-friendly web server to predict changes in binding affinity upon mutations and it is available at https://web.iitm.ac.in/bioinfo2/DeepPPAPredMut/index.html. It accepts the protein sequences in FASTA format, and mutations in a prescribed format, along with protein functional class from the dropdown menu. Upon submission, the server will compute the necessary features and display the ΔΔ*G* (kcal/mol), along with information about whether the given mutation decreases or increases the affinity. For large scale analysis code will be provided on request.

## 4 Conclusion

We have developed DeepPPAPredMut, a robust deep ensemble model to predict the change in binding affinity in protein–protein complexes due to mutation. It exhibits remarkable predictive performance, as evidenced by its high correlation coefficients and minimal MAEs on both training and test datasets. Its reliability is further substantiated through Leave-out-one-complex cross-validation, which simulates real-world conditions. The versatility of the method extends across diverse functional classes of proteins, catering to researchers with varying focus areas. Our use of SHAP-based feature analysis elucidates the critical factors such as structural and network-based features, driving predictions, offering valuable insights into the underlying mechanisms. Moreover, DeepPPAPredMut surpasses existing methods when evaluated on a benchmark dataset and includes a user-friendly web server, enhancing accessibility for researchers.

## Supplementary Material

btae309_Supplementary_Data

## Data Availability

Data available on request.

## References

[btae309-B1] Baek M , DiMaio F, Anishchenko I et al Accurate prediction of protein structures and interactions using a three-track neural network. Science 2021;373:871–6. 34282049 10.1126/science.abj8754 PMC7612213

[btae309-B2] Behbahani YM , Laine E, Carbone A. Deep local analysis deconstructs protein–protein interfaces and accurately estimates binding affinity changes upon mutation. Bioinformatics 2023;39:i544–52. 37387162 10.1093/bioinformatics/btad231 PMC10311296

[btae309-B3] Benedix A , Becker CM, de Groot BL et al Predicting free energy changes using structural ensembles. Nat Methods 2009;6:3–4. 19116609 10.1038/nmeth0109-3

[btae309-B4] Chaudhary P , Naganathan AN, Gromiha MM. Folding RaCe: a robust method for predicting changes in protein folding rates upon point mutations. Bioinformatics 2015;31:2091–7. 25686635 10.1093/bioinformatics/btv091

[btae309-B5] Cukuroglu E , Gursoy A, Nussinov R et al Non-redundant unique interface structures as templates for modeling protein interactions. PLoS One 2014;9:e86738. 24475173 10.1371/journal.pone.0086738 PMC3903793

[btae309-B6] Geng C , Vangone A, Folkers GE et al iSEE: interface structure, evolution, and energy-based machine learning predictor of binding affinity changes upon mutations. Proteins 2019;87:110–9. 30417935 10.1002/prot.25630 PMC6587874

[btae309-B39] Gromiha MM. Protein Bioinformatics: From Sequence to Function. Elsevier Publishers, 2020a.

[btae309-B7] Gromiha MM. Protein interactions. In: Gromiha MM (ed.), Binding Affinity of Protein–Protein Complexes: Experimental Techniques, Databases and Computational Methods, Chap. 4. Singapore: World Scientific, 2020b, 87–108.

[btae309-B8] Gromiha MM. Protein Mutations: Consequences on Structure, Function and Diseases. Singapore: World Scientific, 2024.

[btae309-B9] Gromiha MM , Yugandhar K, Jemimah S. Protein–protein interactions: scoring schemes and binding affinity. Curr Opin Struct Biol 2017;44:31–8. 27866112 10.1016/j.sbi.2016.10.016

[btae309-B10] Guerois R , Nielsen JE, Serrano L. Predicting changes in the stability of proteins and protein complexes: a study of more than 1000 mutations. J Mol Biol 2002;320:369–87. 12079393 10.1016/S0022-2836(02)00442-4

[btae309-B11] Hubbard SJ , Thornton JM. ‘NACCESS’, Computer Program. London: Department of Biochemistry and Molecular Biology, University College, 1993.

[btae309-B12] Jankauskaite J , Jiménez-García B, Dapkunas J et al SKEMPI 2.0: an updated benchmark of changes in protein–protein binding energy, kinetics and thermodynamics upon mutation. Bioinformatics 2019;35:462–9. 30020414 10.1093/bioinformatics/bty635 PMC6361233

[btae309-B13] Jemimah S , Gromiha MM. Exploring additivity effects of double mutations on the binding affinity of protein–protein complexes. Proteins 2018;86:536–47. 29383762 10.1002/prot.25472

[btae309-B14] Jemimah S , Sekijima M, Gromiha MM. ProAffiMuSeq: sequence-based method to predict the binding free energy change of protein–protein complexes upon mutation using functional classification. Bioinformatics 2020;36:1725–30. 31713585 10.1093/bioinformatics/btz829

[btae309-B15] Jemimah S , Yugandhar K, Michael Gromiha M. PROXiMATE: a database of mutant protein–protein complex thermodynamics and kinetics. Bioinformatics 2017;33:2787–8. 28498885 10.1093/bioinformatics/btx312

[btae309-B16] Jumper J , Evans R, Pritzel A et al Highly accurate protein structure prediction with AlphaFold. Nature 2021;596:583–9. 34265844 10.1038/s41586-021-03819-2 PMC8371605

[btae309-B17] Levy ED. A simple definition of structural regions in proteins and its use in analyzing interface evolution. J Mol Biol 2010;403:660–70. 20868694 10.1016/j.jmb.2010.09.028

[btae309-B18] Li G , Pahari S, Murthy AK et al SAAMBE-SEQ: a sequence-based method for predicting mutation effect on protein–protein binding affinity. Bioinformatics 2021;37:992–9. 32866236 10.1093/bioinformatics/btaa761 PMC8128451

[btae309-B19] Li M , Simonetti FL, Goncearenco A et al MutaBind estimates and interprets the effects of sequence variants on protein–protein interactions. Nucleic Acids Res 2016;44:W494–501. 27150810 10.1093/nar/gkw374 PMC4987923

[btae309-B20] Lundberg SM , Nair B, Vavilala MS et al Explainable machine-learning predictions for the prevention of hypoxaemia during surgery. Nat Biomed Eng 2018;2:749–60. 31001455 10.1038/s41551-018-0304-0 PMC6467492

[btae309-B21] Ma BG , Goncearenco A, Berezovsky IN. Thermophilic adaptation of protein complexes inferred from proteomic homology modeling. Structure 2010;18:819–28. 20637418 10.1016/j.str.2010.04.004

[btae309-B22] Manning JR , Jefferson ER, Barton GJ. The contrasting properties of conservation and correlated phylogeny in protein functional residue prediction. BMC Bioinformatics 2008;9:51. 18221517 10.1186/1471-2105-9-51 PMC2267696

[btae309-B23] McDonald IK , Thornton JM. Satisfying hydrogen bonding potential in proteins. J Mol Biol 1994;238:777–93. 8182748 10.1006/jmbi.1994.1334

[btae309-B24] Nikam R , Yugandhar K, Gromiha MM. Deep learning-based method for predicting and classifying the binding affinity of protein–protein complexes. Biochim Biophys Acta Proteins Proteom 2023;1871:140948. Advance online publication. 37567456 10.1016/j.bbapap.2023.140948

[btae309-B25] Nikam R , Yugandhar K, Gromiha MM. DeepBSRPred: deep learning-based binding site residue prediction for proteins. Amino Acids 2022;55:1305–16. 36574037 10.1007/s00726-022-03228-3

[btae309-B26] Nishi H , Tyagi M, Teng S et al Cancer missense mutations alter binding properties of proteins and their interaction networks. PLoS One 2013;8:e66273. 23799087 10.1371/journal.pone.0066273 PMC3682950

[btae309-B27] Pahari S , Li G, Murthy AK et al SAAMBE-3D: predicting effect of mutations on protein–protein interactions. Int J Mol Sci 2020;21:2563. 32272725 10.3390/ijms21072563 PMC7177817

[btae309-B28] Pandey M , Gromiha MM. MutBLESS: a tool to identify disease-prone sites in cancer using deep learning. Biochim Biophys Acta Mol Basis Dis 2023;1869:166721. 37105446 10.1016/j.bbadis.2023.166721

[btae309-B29] Pedregosa F , Varoquaux G, Gramfort A et al Scikit-learn: machine learning in Python. J Mach Learn Res 2011;12:2825–30.

[btae309-B30] Petukh M , Kucukkal TG, Alexov E. On human disease-causing amino acid variants: statistical study of sequence and structural patterns. Hum Mutat 2015;36:524–34. 25689729 10.1002/humu.22770 PMC4409542

[btae309-B31] Rodrigues CHM , Myung Y, Pires DEV et al mCSM-PPI2: predicting the effects of mutations on protein–protein interactions. Nucleic Acids Res 2019;47:W338–44. 31114883 10.1093/nar/gkz383 PMC6602427

[btae309-B32] Schymkowitz J , Borg J, Stricher F et al The FoldX web server: an online force field. Nucleic Acids Res 2005;33:W382–8. 15980494 10.1093/nar/gki387 PMC1160148

[btae309-B33] Valdar WS. Scoring residue conservation. Proteins 2002;48:227–41. 12112692 10.1002/prot.10146

[btae309-B34] Varadi M , Anyango S, Deshpande M et al AlphaFold protein structure database: massively expanding the structural coverage of protein-sequence space with high-accuracy models. Nucleic Acids Res 2022;50:D439–44. 34791371 10.1093/nar/gkab1061 PMC8728224

[btae309-B35] Xiong P , Zhang C, Zheng W et al BindProfX: assessing mutation-induced binding affinity change by protein interface profiles with pseudo-counts. J Mol Biol 2017;429:426–34. 27899282 10.1016/j.jmb.2016.11.022 PMC5963940

[btae309-B36] Yugandhar K , Gromiha MM. Feature selection and classification of protein–protein complexes based on their binding affinities using machine learning approaches. Proteins2014a;82:2088–96. 24648146 10.1002/prot.24564

[btae309-B37] Yugandhar K , Gromiha MM. Protein–protein binding affinity prediction from amino acid sequence. Bioinformatics2014b;30:3583–9. 25172924 10.1093/bioinformatics/btu580

[btae309-B38] Zhang N , Chen Y, Lu H et al MutaBind2: predicting the impacts of single and multiple mutations on protein–protein interactions. iScience 2020;23:100939. 32169820 10.1016/j.isci.2020.100939 PMC7068639

