# Supplementary material for: DeepPPAPredMut: deep ensemble method for predicting the binding affinity change in protein–protein complexes upon mutation
Source: Bioinformatics. 2024 May 8;40(5):btae309. doi: 10.1093/bioinformatics/btae309 (PMC11112046; doi:10.1093/bioinformatics/btae309)
Supplement: btae309_Supplementary_Data [file btae309_supplementary_data.docx]

**Supplementary Information**

**DeepPPAPredMut: deep ensemble method for predicting the binding affinity change in protein-protein complexes upon mutation**

**Rahul Nikam^1^, Sherlyn Jemimah^1,2^ and M. Michael Gromiha^1,3, *^**

^1^Department of Biotechnology, Bhupat and Jyoti Mehta School of Biosciences, Indian Institute of Technology Madras, Chennai 600036, Tamil Nadu, India

^2^Department of Biomedical Engineering, Khalifa University, Abu Dhabi, United Arab Emirates.

^3^Department of Computer Science, Tokyo Institute of Technology, Yokohama, Japan.

*To whom correspondence should be addressed.

Tel: +914422574138

Fax: +91 44 2257 4102

E-mail: gromiha@iitm.ac.in

**ABBREVIATIONS**

| ASA | Accessible surface area |
| --- | --- |
| PSSM | Position-Specific Scoring Matrix |
| Ag-Ab | Antigen-antibody complex |
| EI | Enzyme-inhibitor complex |
| GC | G-protein complex |
| MS | Miscellaneous complex |
| RC | Receptor complex |
| OE | Other enzyme complex |
| R | Pearson’s correlation |
| MAE | Mean absolute error |
| PPIs | Protein-protein interactions |
| DNNs | Deep Neural Networks |
| CNNs | Convolutional Neural Networks |
| RNNs | Recurrent Neural Networks |
| LOOC-CV | Leave-out-one complex cross-validation |


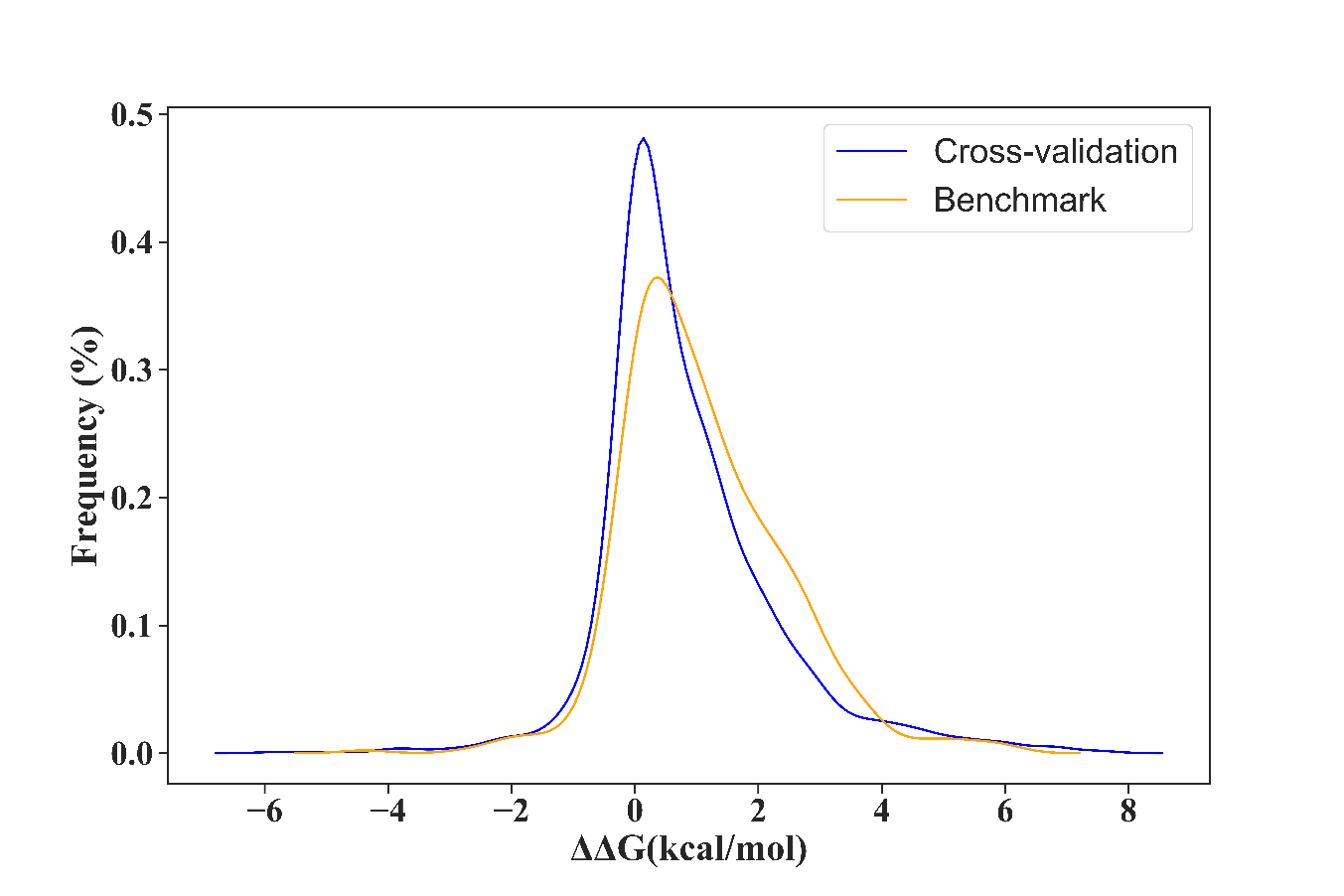


**Supplementary Figure S1:** Distribution of ΔΔG in cross-validation and benchmark datasets.

**
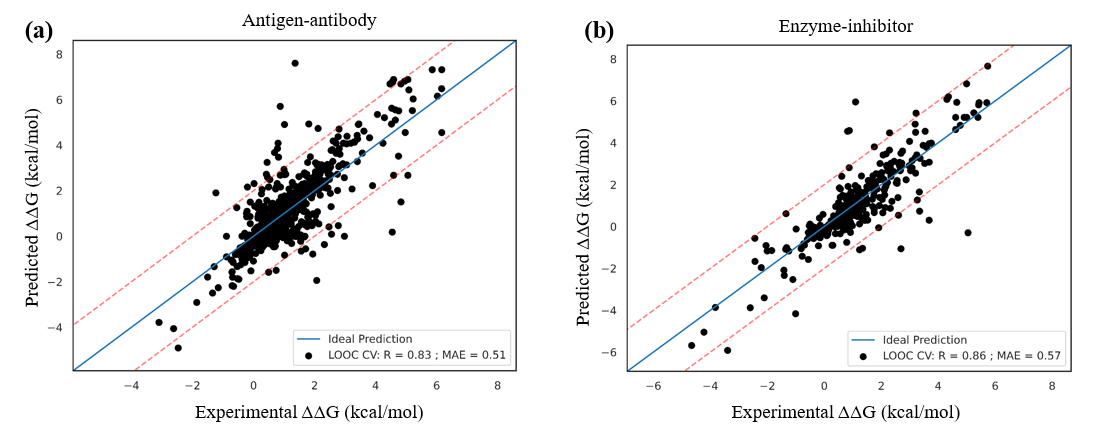
**

**
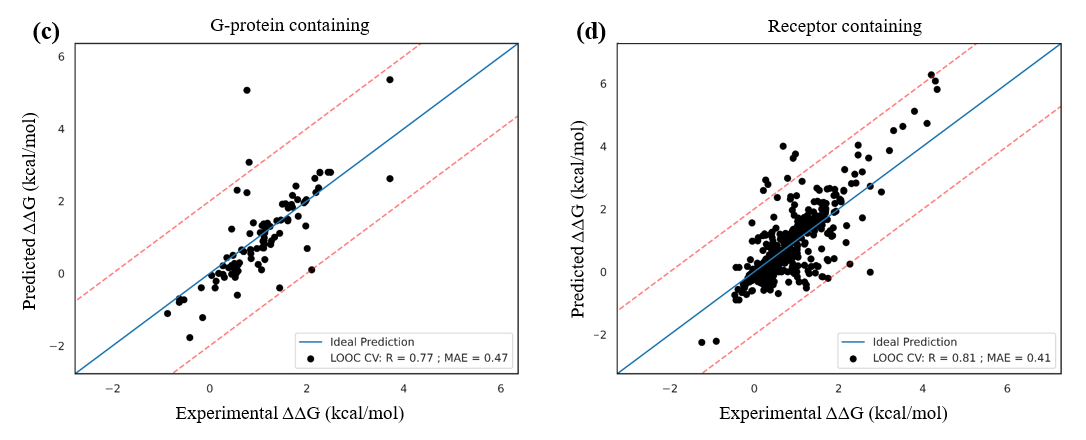
**

**
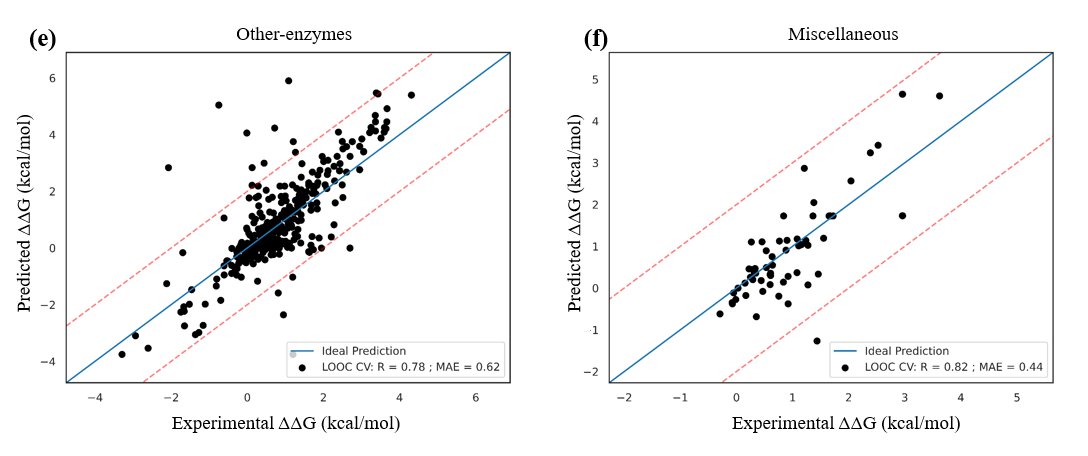
**

**Supplementary Figure S2:** Prediction performance on individual functional class on LOOC cross validation.


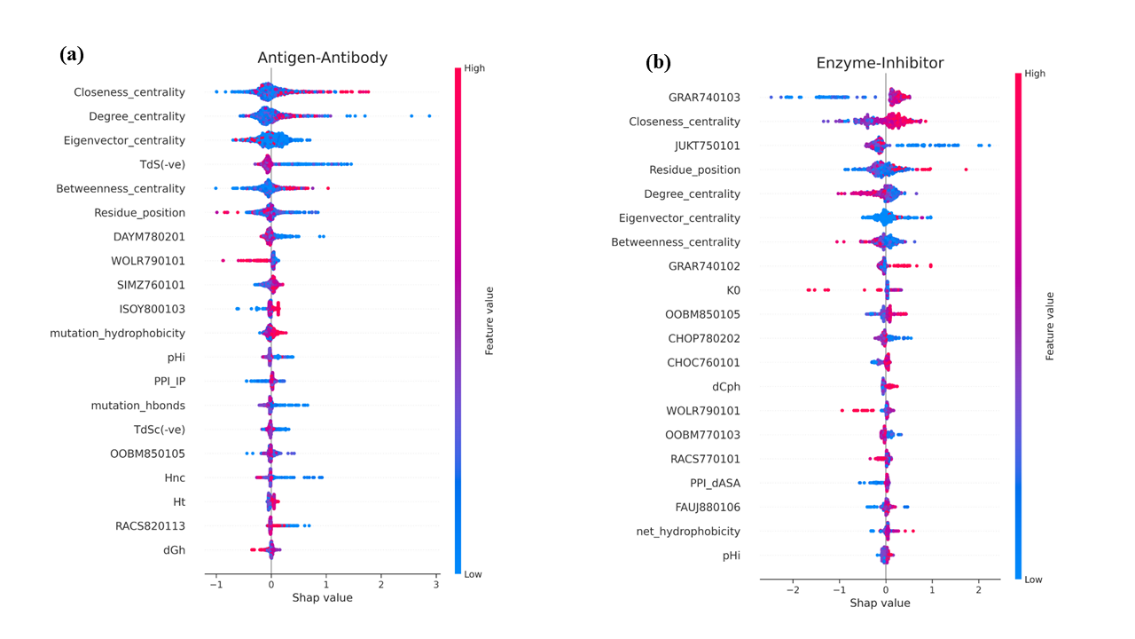

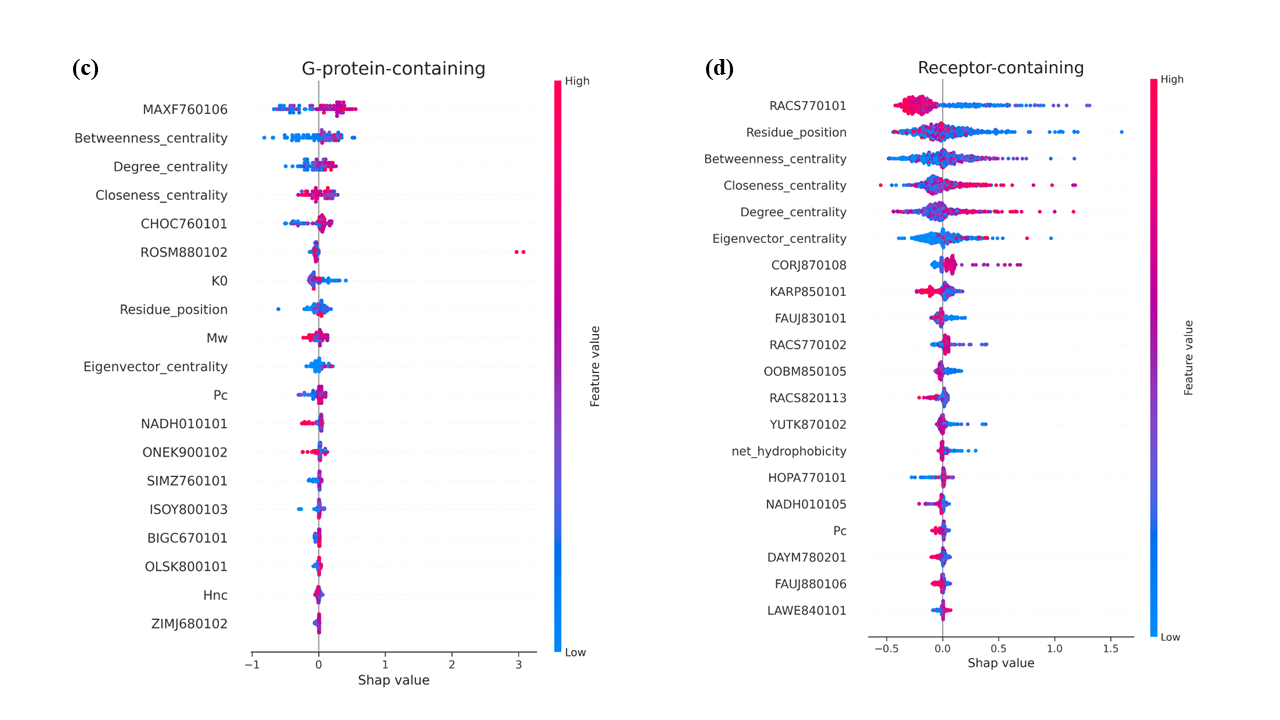

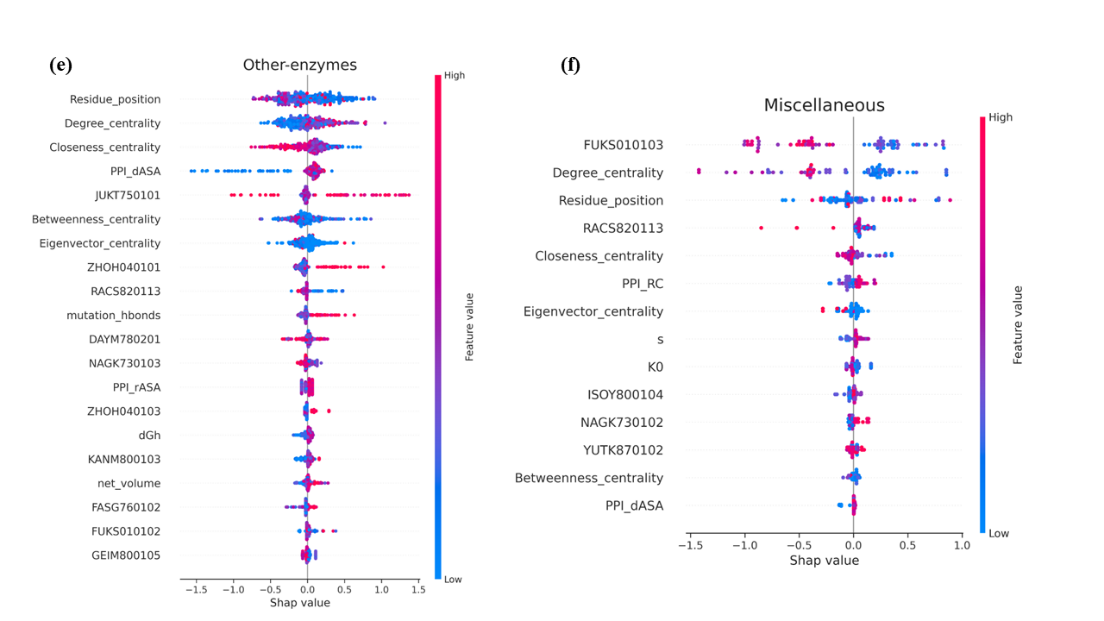


**Supplementary Figure S3:** SHAP plots showing the contribution of each feature selected in each functional class: (a) antigen-antibody (b) enzyme-inhibitor (c) G-protein-containing (d) receptor-containing (e) other-enzymes and (f) miscellaneous. Description of each feature is given in **Supplementary Table S1**

**Supplementary Table S1**

Description features used in the present study*

| **No** | **Property name** | **Description** |
| --- | --- | --- |
|  | -TdS | Unfolding entropy change (Gromiha, 2005) |
|  | -TdSc | Unfolding entropy change of chain (Gromiha, 2005) |
|  | -TdSh | Unfolding entropy change of hydration (Gromiha, 2005) |
|  | ARGP820101 | Hydrophobicity index (Argos et al., 1982) |
|  | *BIGC670101* | *Residue volume (Bigelow 1967)* |
|  | BIOV880102 | Information value for accessibility; average fraction 23%(Biou et al., 1988) |
|  | Bl | Bulkiness (Gromiha, 2005) |
|  | BURA740102 | Normalized frequency of extended structure (Burgess et al., 1974) |
|  | CHAM820101 | Polarizability parameter (Charton-Charton, 1982) |
|  | *CHOC760101* | *Residue accessible surface area in tripeptide (Chothia 1976)* |
|  | *CHOP780202* | *Normalized frequency of beta-sheet (Chou-Fasman 1978b)* |
|  | CHOP780204 | Normalized frequency of N-terminal helix (Chou-Fasman, 1978b) |
|  | CORJ870106 | ALTLS index (Cornette et al., 1987) |
|  | *CORJ870108* | *TOTLS index (Cornette et al. 1987)* |
|  | *DAYM780201* | *Relative mutability (Dayhoff et al. 1978b)* |
|  | *dCph* | *Unfolding hydration heat capacity change (Gromiha, 2005)* |
|  | *Degree, Closeness, Eigenvector centrality* | *Graph based properties centrality measures* |
|  | *dGh* | *Gibbs free energy change of hydration for unfolding (Gromiha, 2005)* |
|  | EISD860101 | Solvation free energy (Eisenberg-McLachlan, 1986) |
|  | *FASG760102* | *Melting point (Fasman 1976)* |
|  | *FAUJ830101* | *Hydrophobic parameter pi (Fauchere-Pliska 1983)* |
|  | FAUJ830101 | Hydrophobic parameter pi (Fauchere-Pliska, 1983) |
|  | FAUJ880101 | Graph shape index (Fauchere et al., 1988) |
|  | *FAUJ880106* | *STERIMOL maximum width of the side chain (Fauchere et al. 1988)* |
|  | *FUKS010102* | *Surface composition of amino acids in intracellular proteins of mesophiles (percent) (Fukuchi-Nishikawa 2001)* |
|  | *FUKS010103* | *Surface composition of amino acids in extracellular proteins of mesophiles (percent) (Fukuchi-Nishikawa 2001)* |
|  | *GEIM800105* | *Beta-strand indices (Geisow-Roberts 1980)* |
|  | GEIM800108 | Aperiodic indices (Geisow-Roberts, 1980) |
|  | *GRAR740102* | *Polarity (Grantham 1974)* |
|  | *GRAR740103* | *Volume (Grantham 1974)* |
|  | *Hnc* | *Normalized consensus hydrophobicity* |
|  | *HOPA770101* | *Hydration number (Hopfinger 1971)* |
|  | Hp | Surrounding hydrophobicity (Gromiha, 2005) |
|  | *Ht* | *Thermodynamic transfer hydrophobicity* |
|  | *ISOY800103* | *Normalized relative frequency of bend (Isogai et al. 1980)* |
|  | *ISOY800104* | *Normalized relative frequency of bend R (Isogai et al. 1980)* |
|  | JOND750101 | Hydrophobicity (Jones, 1975) |
|  | *JUKT750101* | *Amino acid distribution (Jukes et al. 1975)* |
|  | *K0* | *Compressibility* |
|  | KANM800102 | Average relative probability of beta-sheet (Kanehisa-Tsong, 1980) |
|  | *KANM800103* | *Average relative probability of inner helix (Kanehisa-Tsong 1980)* |
|  | *KARP850101* | *Flexibility parameter for no rigid neighbors (Karplus-Schulz 1985)* |
|  | KRIW710101 | Side chain interaction parameter (Krigbaum-Rubin, 1971) |
|  | KRIW790101 | Side chain interaction parameter (Krigbaum-Komoriya, 1979) |
|  | *LAWE840101* | *Transfer free energy CHP/water (Lawson et al. 1984)* |
|  | LEVM760101 | Hydrophobic parameter (Levitt, 1976) |
|  | LIFS790101 | Conformational preference for all beta-strands (Lifson-Sander, 1979) |
|  | LIFS790103 | Conformational preference for antiparallel beta-strands (Lifson-Sander, 1979) |
|  | MANP780101 | Average surrounding hydrophobicity (Manavalan-Ponnuswamy, 1978) |
|  | MAXF760102 | Normalized frequency of extended structure (Maxfield-Scheraga, 1976) |
|  | *MAXF760106* | *Normalized frequency of alpha region (Maxfield-Scheraga 1976)* |
|  | MIYS850101 | Effective partition energy (Miyazawa-Jernigan, 1985) |
|  | MIYS990101 | Relative partition energies derived by the Bethe approximation (Miyazawa-Jernigan, 1999) |
|  | Mu | Refractive index (Gromiha, 2005) |
|  | *Mutation hbonds* | *Change in hydrogen bonds* |
|  | *Mutation hydrophobicity* | *Hydrophobicity of mutated residue* |
|  | *Mw* | *Molecular weight* |
|  | *NADH010101* | *Hydropathy scale based on self-information values in the two-state model (5% accessibility) (Naderi-Manesh et al. 2001)* |
|  | NADH010102 | Hydropathy scale based on self-information values in the two-state model (9% Accessibility, Naderi-Manesh et al., 2001) |
|  | NADH010104 | Hydropathy scale based on self-information values in the two-state model (20% Accessibility, Naderi-Manesh et al., 2001) |
|  | *NADH010105* | *Hydropathy scale based on self-information values in the two-state model (25% accessibility) (Naderi-Manesh et al. 2001)* |
|  | *NAGK730102* | *Normalized frequency of bata-structure (Nagano 1973)* |
|  | *Net hydrophobicity* | *Change in hydrophobicity* |
|  | *Net volume* | *Change in volume* |
|  | NISK860101 | 14 A contact number (Nishikawa-Ooi, 1986) |
|  | Nl | Average long-range contacts (Gromiha, 2005) |
|  | *OLSK800101* | *Average internal preferences (Olsen 1980)* |
|  | *ONEK900102* | *Helix formation parameters (delta delta G) (O'Neil-DeGrado 1990)* |
|  | *OOBM770103* | *Long range non-bonded energy per atom (Oobatake-Ooi 1977)* |
|  | OOBM770103 | Long range non-bonded energy per atom (Oobatake-Ooi, 1977) |
|  | OOBM850103 | Optimized transfer energy parameter (Oobatake et al., 1985) |
|  | *OOBM850105* | *Optimized side chain interaction parameter (Oobatake et al. 1985)* |
|  | P | Polarity (Gromiha, 2005) |
|  | Pb | Beta-helical tendency (Gromiha, 2005) |
|  | *Pc* | *Coil tendency* |
|  | *pHi* | *Isoelectric point* |
|  | pHi | Isoelectric point (Gromiha, 2005) |
|  | pK' | Equilibrium constant with reference to the ionization property of COOH group (Gromiha, 2005) |
|  | PONP800102 | Average gain in surrounding hydrophobicity (Ponnuswamy et al., 1980) |
|  | PONP800103 | Average gain ratio in surrounding hydrophobicity (Ponnuswamy et al., 1980) |
|  | PONP800107 | Accessibility reduction ratio (Ponnuswamy et al., 1980) |
|  | *PPI_dASA, PPI_rASA, PPI_RC* | *Interface properties defined by Jemimah et al. 2020* |
|  | Ra | Solvent accessible reduction ratio (Gromiha, 2005) |
|  | *RACS770101* | *Average reduced distance for C-alpha (Rackovsky-Scheraga 1977)* |
|  | RACS770102 | Average reduced distance for side chain (Rackovsky-Scheraga, 1977) |
|  | *RACS820113* | *Value of theta(i) (Rackovsky-Scheraga 1982)* |
|  | *Residue position* | *Mutation position in the protein* |
|  | Rf | Chromatographic index (Gromiha, 2005) |
|  | ROBB760105 | Information measure for extended (Robson-Suzuki, 1976) |
|  | *ROSM880102* | *Side chain hydropathy corrected for solvation (Roseman 1988)* |
|  | *s* | *Shape (position of branch point in a side-chain)* |
|  | *SIMZ760101* | *Transfer free energy (Simon 1976) Cited by Charton-Charton (1982)* |
|  | *TdS(-ve)* | *Unfolding entropy change* |
|  | *TdSc(-ve)* | *Unfolding entropy change of chain* |
|  | V0 | Partial-specific volume (Gromiha, 2005) |
|  | WOEC730101 | Polar requirement (Woese, 1973) |
|  | *WOLR790101* | *Hydrophobicity index (Wolfenden et al. 1979)* |
|  | *YUTK870102* | *Unfolding Gibbs energy in water pH9.0 (Yutani et al. 1987)* |
|  | ZHOH040101 | The stability scale from the knowledge-based atom-atom potential (Zhou-Zhou,††2004) |
|  | *ZHOH040103* | *Buriability (Zhou-Zhou 2004)* |
|  | ZIMJ680101 | Hydrophobicity (Zimmerman et al., 1968) |
|  | *ZIMJ680102* | *Bulkiness (Zimmerman et al. 1968)* |
|  | ZIMJ680105 | RF rank (Zimmerman et al., 1968) |

Features selected in prediction models are shown in italics.

**Supplementary Table S2**

Performance using different machine learning methods

| **Method** | **Correlation** | **MAE (kcal/mol)** |
| --- | --- | --- |
| Basic Linear Regression | 0.70 | 0.65 |
| Decision Tree | 0.65 | 0.70 |
| k-Nearest Neighbors | 0.60 | 0.75 |
| Neural Network | 0.72 | 0.63 |
| SVM Regression | 0.68 | 0.68 |
| **Random Forest** | **0.83** | **0.51** |

**Supplementary Table S3**

Prediction performance using two different scenarios for cross validations

| **Functional class** | **r1** | **r2** | **MAE1 kcal/mol** | **MAE2 kcal/mol** |
| --- | --- | --- | --- | --- |
| Antigen-antibody | 0.85 | 0.81 | 0.52 | 0.53 |
| Enzyme-inhibitor | 0.90 | 0.85 | 0.56 | 0.57 |
| G-protein-containing | 0.78 | 0.78 | 0.48 | 0.47 |
| Receptor-containing | 0.93 | 0.81 | 0.40 | 0.43 |
| Other-enzyme containing | 0.82 | 0.81 | 0.60 | 0.58 |
| Miscellaneous | 0.88 | 0.85 | 0.39 | 0.39 |
| Overall | 0.86 | 0.82 | 0.49 | 0.50 |

**Scenario 1**: r1 and MAE1: cross validations are performed randomly at the level of variations, allowing two different substitutions occurring at the same site to be placed in the training and testing set, respectively.

**Scenario 2**: r2 and MAE2: splits are performed such that mutations occurring at the same site are all confined either in training or testing sets.
